# A deep learning model using chest X-ray for identifying TB and NTM-LD patients: a cross-sectional study

**DOI:** 10.1186/s13244-023-01395-9

**Published:** 2023-04-15

**Authors:** Chia-Jung Liu, Cheng Che Tsai, Lu-Cheng Kuo, Po-Chih Kuo, Meng-Rui Lee, Jann-Yuan Wang, Jen-Chung Ko, Jin-Yuan Shih, Hao-Chien Wang, Chong-Jen Yu

**Affiliations:** 1 Department of Internal Medicine, National Taiwan University Hospital, Hsin-Chu Branch, Hsinchu, Taiwan; 2 Graduate Institute of Clinical Medicine, College of Medicine, National Taiwan University, Taipei, Taiwan; 3 Department of Computer Science, University of North Carolina at Chapel Hill, Chapel Hill, NC USA; 4 Department of Internal Medicine, National Taiwan University Hospital, #7, Zhongshan South Rd., Zhongzheng Dist., Taipei, 100226 Taiwan; 5 Department of Computer Science, National Tsing Hua University, No. 101, Kuang Fu Rd, Sec.2, Hsinchu, 300044 Taiwan; 6 Department of Medicine, National Taiwan University Cancer Center, Taipei, Taiwan

**Keywords:** Nontuberculous mycobacteria, Tuberculosis, Chest radiography, Deep learning, Artificial intelligence

## Abstract

**Background:**

Timely differentiating between pulmonary tuberculosis (TB) and nontuberculous mycobacterial lung disease (NTM-LD), which are radiographically similar, is important because infectiousness and treatment differ. This study aimed to evaluate whether artificial intelligence could distinguish between TB or NTM-LD patients by chest X-rays (CXRs) from suspects of mycobacterial lung disease.

**Methods:**

A total of 1500 CXRs, including 500 each from patients with pulmonary TB, NTM-LD, and patients with clinical suspicion but negative mycobacterial culture (Imitator) from two hospitals, were retrospectively collected and evaluated in this study. We developed a deep neural network (DNN) and evaluated model performance using the area under the receiver operating characteristic curves (AUC) in both internal and external test sets. Furthermore, we conducted a reader study and tested our model under three scenarios of different mycobacteria prevalence.

**Results:**

Among the internal and external test sets, the AUCs of our DNN model were 0.83 ± 0.005 and 0.76 ± 0.006 for pulmonary TB, 0.86 ± 0.006 and 0.64 ± 0.017 for NTM-LD, and 0.77 ± 0.007 and 0.74 ± 0.005 for Imitator. The DNN model showed higher performance on the internal test set in classification accuracy (66.5 ± 2.5%) than senior (50.8 ± 3.0%, *p* < 0.001) and junior pulmonologists (47.5 ± 2.8%, *p* < 0.001). Among different prevalence scenarios, the DNN model has stable performance in terms of AUC to detect TB and mycobacterial lung disease.

**Conclusion:**

DNN model had satisfactory performance and a higher accuracy than pulmonologists on classifying patients with presumptive mycobacterial lung diseases. DNN model could be a complementary first-line screening tool.

**Supplementary Information:**

The online version contains supplementary material available at 10.1186/s13244-023-01395-9.

## Key points

- The DNN model showed significantly higher classification accuracy compared to pulmonologists.
- The DNN model has stable performance in different mycobacteria prevalence scenarios.
- DNN model could be a screening tool for mycobacterial lung diseases.

**Supplementary Information:**

The online version contains supplementary material available at 10.1186/s13244-023-01395-9.

## Background

Pulmonary diseases caused by mycobacteria, including *Mycobacterium tuberculosis* and non-tuberculous mycobacteria (NTM), can cause a significant impact on human health [1]. Tuberculosis (TB) remains one of the most important infectious diseases worldwide, leading to significant mortality and morbidity [2]. However, a paradoxical trend of decreasing TB and increasing NTM-lung disease (NTM-LD) patients was found in many countries [3]. Simultaneously, several reports have found the NTM-LD prevalence was much higher than expectation and even more than pulmonary TB [4–6].

Timely differentiating between TB and NTM lung disease is crucial because therapeutic regimens differ between these two diseases, and it is necessary to conduct isolation and contact investigations for patients with pulmonary TB [4, 7, 8]. Distinguishing pulmonary TB from NTM-LD, however, remains challenging because of considerable overlap in the clinical and radiographic findings even if chest computed tomography (CT) is performed [9]. Nevertheless, existing diagnostic tools have some undesirable weaknesses. For instance, the turnaround time of mycobacterial culture may take up to several weeks [1]. While molecular techniques including the cartridge-based nucleic acid amplification test or line probe assays are less time-consuming, they are more expensive and likely to struggle with paucibacillary specimens [10]. Thus, an efficient and low-cost tool to distinguish between pulmonary TB and NTM-LD is demanded.

In recent years, fast evolution of artificial intelligence has demonstrated promising results in the detection of pulmonary TB on chest X-ray (CXR) [11, 12]. However, these reports only demonstrated machines’ utility on classification between TB and relatively healthy patients. The previous results therefore may deviate from clinicians’ experience in which they need to make hard diagnosis between TB, NTM-LD, and suspects of mycobacterial lung disease who were later excluded because of negative mycobacterial surveillance.

Hence, in this study, we aim to provide evidence to close this gap. We recruited patients with TB, NTM-LD, and other presumptive mycobacterial lung diseases, and develop deep neural network (DNN) models to distinguish them. We also carefully estimate models’ performance in environments with different mycobacteria prevalence and evaluate the application limitation of not including NTM-LD in the training cohort.

## Methods

### Study design and data collection

This study was conducted in two hospitals. To investigate the performance of the DNN model in patients with presumptive mycobacterial lung disease, we enrolled patients with pulmonary TB, NTM-LD, or presumptive mycobacterial lung diseases who have at least three consecutive negative sputum cultures for mycobacteria (imitators of mycobacterial lung diseases, the Imitator group). The pulmonary TB was diagnosed based on mycobacterial cultures from respiratory specimens. The diagnosis of NTM-LD was made for those that had met the clinical, radiographic and microbiologic criteria, according to the current NTM-LD guideline [13].

### CXR datasets

**Fig. 1:**
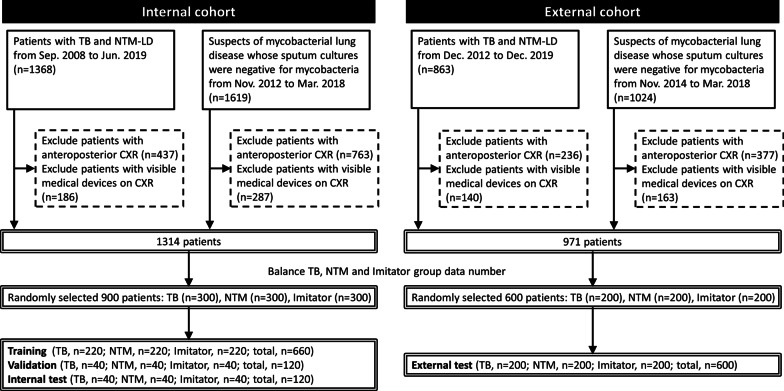
Flowchart of dataset establishment from patients with presumptive mycobacterial lung diseases is presented. In brief, a total of 2987 and 1887 patients with tuberculosis (TB), non-tuberculous mycobacteria lung disease (NTM-LD) or suspicious of mycobacterial lung disease whose sputum cultures were negative for mycobacteria (Imitator) were identified in the internal and external cohort, respectively. After excluding patients with anteroposterior chest X-ray (CXR) or with visible medical devices on CXR, 1314 and 971 patients were enrolled in the internal and external cohort. Then, we randomly and equally collected 300 patients for each TB/NTM-LD/Imitator group in the internal cohort, and 200 patients for each TB/NTM-LD/Imitator group in the external cohort to ensure our model could fairly learn from each diagnosis. Finally, 900 patients in the internal cohort were randomly assigned to one of the three datasets: training, internal validation and internal test

CXRs used in this study were stored in digital films for clinical use from patients who visited these two hospitals (internal and external cohort) from September 2008 to December 2019. Figure 1 shows the flowchart of dataset creation. The interval between a selected CXR and the date of respiratory specimen for the mycobacterial study was restrained to less than one month. The CXRs with anteroposterior views or visible medical devices were excluded. Two experienced pulmonologists, who were blinded to the clinical information, evaluated the characteristics of each CXR respectively, including the pattern (consolidation, cavitation, pleural effusion or others) and extent (multifocal or focal) according to standard protocol [14]. In cases of discrepancy, a final decision was achieved through consensus.

To ensure that the model could fairly learn from each diagnosis, we randomly and equally collected 300 CXRs for each TB/NTM-LD/Imitator group and 200 CXRs for each TB/NTM-LD/Imitator group from the internal and external cohort, respectively. A total of 900 CXRs in the internal cohort were randomly assigned to one of the three datasets: training (*n* = 220 for each TB/NTM-LD/Imitator group), internal validation (*n* = 40 for each TB/NTM-LD/Imitator group), and internal test (*n* = 40 for each TB/NTM-LD/Imitator group). In the external cohort, 600 CXRs (*n* = 200 for each TB/NTM-LD/Imitator group) were kept untouched until testing the trained model (external test). Each image was then resized to 320 by 320 pixels before feeding the DNN model.

We also included CXRs from two public databases for model pretraining. In the end, 248,285 CXR images from MIMIC-CXR [15] and 189,892 CXR images from CheXpert [16] databases were recruited. Similarly, images from MIMIC and CheXpert contain labels of 14 common radiographic observations, which do not explicitly include TB, NTM-LD, or Imitator.

### Development of the DNN model

**Fig. 2:**
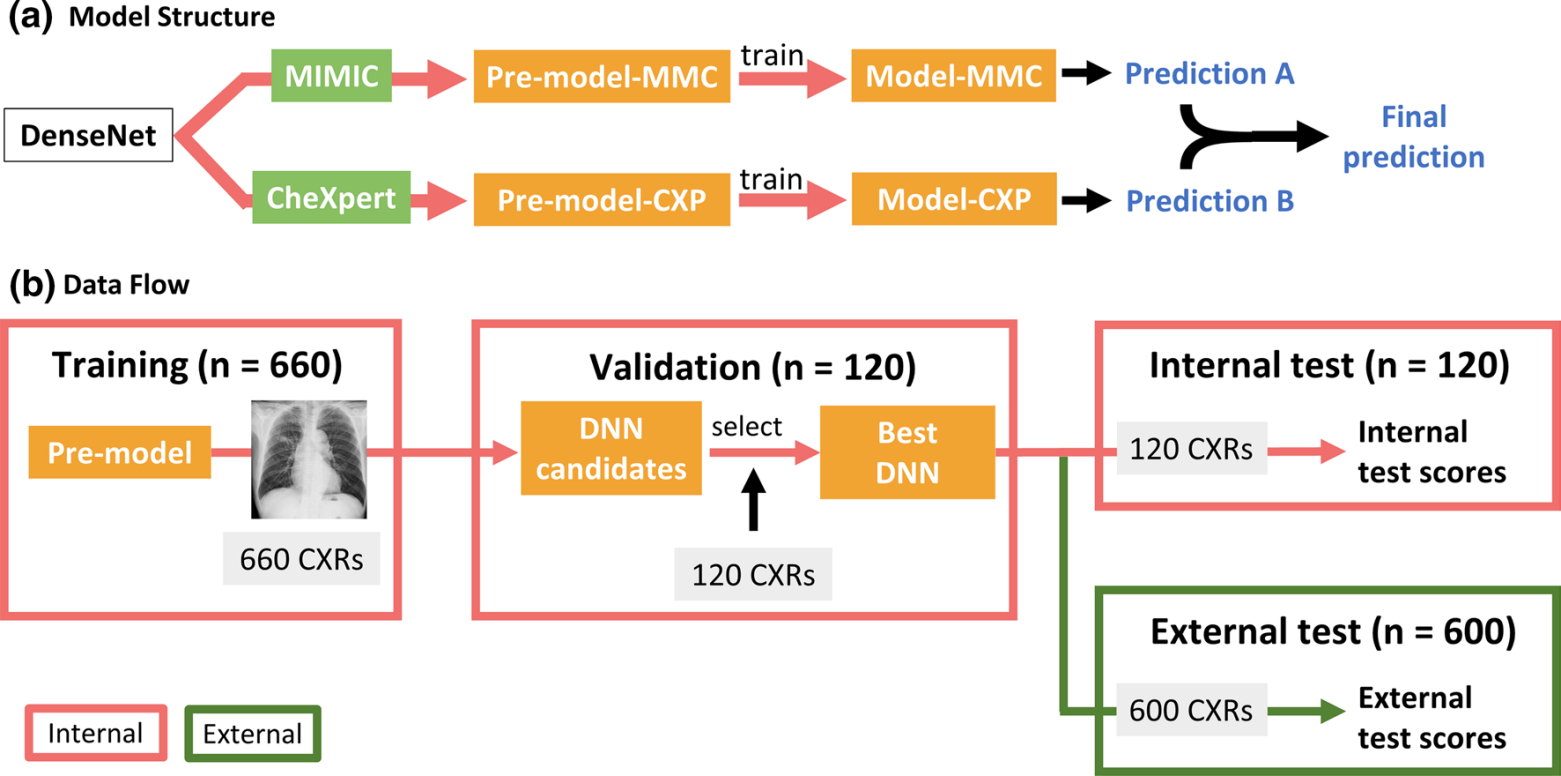
**a** presents the architecture of the deep neural network (DNN). This ensemble learning framework relies on pretraining two DenseNet models separately on two large public datasets (MIMIC and CheXpert) and fine-tuning them on our in-house mycobacterial datasets. **b** illustrates the developing process of our DNN and the data flow. The validation set was used to select the best DNN models. Patients in the internal test set came from the same data distribution as the internal training set. Patients in the external test set came from a different data distribution, bringing more challenges to the model

To develop DNN models, we used the Tensorflow and Keras modules in python and selected the built-in DenseNet121 [17] structure as our DNN’s backbone. All computation process was completed on Google Cloud Platform. Figure 2A summarizes the architecture of our final DNN. Firstly, CXRs from MIMIC (MMC) and CheXpert (CXP) were separately used to pre-train the DenseNet backbone. The result DenseNets were called pre-model-MMC and pre-model-CXP respectively. We then froze the encoders of the two pre-models and replaced their decision layer with a multi-layer perceptron consisting of two 512-neuron layers. The two pre-models were then trained on our in-house datasets to recognize TB, NTM-LD, and Imitator. They were finalized as model-MMC and model-CXP respectively. A detailed discussion about this transfer learning process can be found in Additional file 1: Appendix A. At prediction phase, we utilized these two models as components and applied ensemble learning to establish our final DNN model. Namely, to produce the final predictions, the output predictions from the two models were weight-averaged based on models’ performance on the training set. More details about pre-training can be found in Additional file 1: Appendix A. Figure 2B details the data (CXR) flow. After training, the internal validation set was used to evaluate whether the training result was satisfactory and then select the best-performing model (see Additional file 1: Appendix B). The 120 CXRs in the internal test set were simultaneously used to test our model and the participating physicians.

### Model performance and reader study

In-house DNN performance was assessed using 120 CXRs in the internal test set whereas the 600 CXRs in the external test set were used to evaluate the external generalizability of the model (Fig. 2B). We used the one-versus-others type of area under the receiver operating characteristic curve (AUC) to assess models’ performance.

In parallel, we conducted a reader study to compare the performance between our DNN model and pulmonologists when making the hard radiological diagnosis from similar mycobacterial diseases. We recruited 12 board-certified pulmonologists including 6 senior physicians with more than 10 years of experience on managing patients with mycobacterial lung diseases and 6 junior physicians with less than 10 years of experience from 9 hospitals. These physicians, who were blinded to the clinical information, were asked to independently assess the same 120 CXR in the internal test set and make a diagnosis among TB, NTM-LD, or Imitator based on CXR findings.

### Evaluation of the model as a screening tool in different mycobacteria prevalence

To evaluate model’s potential of assisting in mycobacteria screening in the real world, we simulated three scenarios with different disease prevalence: low TB burden (3.3% TB, 16.7% NTM-LD, 80% Imitator), medium TB burden (5% TB, 5% NTM-LD, 90% Imitator), and high TB burden (12.5% TB, 4.2% NTM-LD, 83.3% Imitator). For each scenario, we followed the standard Bootstrap protocol [18] and sampled 120 patients with replacement from the internal test set according to the given disease prevalence and repeated the entire process for 100 times. The DNN model was required to complete two tasks. The “TB test” required the model to determine whether a patient is a TB patient from NTM-LD and Imitator. The “Mycobacteria test” required the model to differentiate mycobacterial lung disease including TB and NTM-LD from Imitator. We calculated AUC, sensitivity, specificity, positive predictive value, negative predictive value to represent DNN’s capability as a screening tool. Furthermore, “reduced further test” computes percentage of patients that this screening tool saves from requesting further examination including mycobacterial culture or nucleic acid amplification test for *Mycobacterium tuberculosis* complex (TB-PCR). “Number needs to screen” represents the number of mycobacterial cultures required to catch a confirmed case in the “positive” group selected by the DNN. Lastly, “Misclassified” denotes the number incorrectly predicted by the model.

### Evaluation of DNN models with and without NTM-LD in model development

A distinct feature of this study is our inclusion of NTM-LD patients for developing models. To illustrate its importance, we developed an additional counterpart model (DNN model analogue) using only TB and Imitator patients in the training set, which is a commonly used patient configuration for training TB DNN model in the past research [19]. Then, we repeated the “TB test” and “Mycobacteria test” using this DNN model analogue and compared the performance to our DNN’s under three clinical scenarios with low, medium and high TB burden as mentioned above.

### Statistical analysis

All variables were expressed as numbers (percentages) or mean ± standard deviation as appropriate. The one-way analysis of variance (ANOVA) was used to analyze intergroup differences for continuous variables. The chi-square test was used for categorical variables. To evaluate DNN performance and match the number of recruited doctors, we repeated the whole model training process 12 times with different initial random seeds and calculated standard deviation. For the human–machine comparison, we used the diagnosis accuracy and confusion matrices to present the performance of our DNN and pulmonologists. All *p* values were two-sided and statistical significance was set at *p* < 0.05.

### Model visualization

To better understand how our model differentiated between these three confusing groups, we visualized our model’s attention by using Grad-cam [20]. We presented representative cases of the resulting heatmap among the TB, NTM-LD and Imitator group.

## Results

### Clinical characteristics of the enrolled patients

The clinical characteristics of enrolled patients in the internal training set, internal test set, and external test set are listed in Table 1. The characteristics of patients in internal validation set are described in Additional file 1: Appendix D. In comparison of patients with TB, NTM-LD, and Imitator, patients with TB consisted of a higher percentage of males in all datasets. Regarding CXR pattern, patients with TB had a higher rate of pleural effusion. By contrast, patients with NTM-LD were more likely to have bronchiectasis.

**Table 1:** Demographics, microbiology and radiology data of patients with presumptive mycobacterial lung diseases

	Internal cohort, training (*n* = 660)				Internal cohort, internal test (*n* = 120)				External cohort, external test (*n* = 600)			
	TB	NTM-LD	Imitator	*p*	TB	NTM-LD	Imitator	*p*	TB	NTM-LD	Imitator	*p*
	(*N* = 220)	(*N* = 220)	(*N* = 220)		(*n* = 40)	(*n* = 40)	(*n* = 40)		(*n* = 200)	(*n* = 200)	(*n* = 200)	
Age (years)*	64.3 ± 18.2	67.7 ± 12.7	66.2 ± 15.7	0.075	66.8 ± 18.7	70.1 ± 10.2	62.0 ± 16.1	0.063	55.0 ± 20.5	65.9 ± 15.4	65.8 ± 12.4	< 0.001
Male, *n* (%)	143 (65%)	104 (47%)	101 (46%)	< 0.001	29 (73%)	19 (48%)	16 (40%)	0.010	140 (70%)	110 (55%)	83 (42%)	< 0.001
*Acid-fast smear*				< 0.001				< 0.001				< 0.001
High-grade positive (3, 4)	53 (24%)	34 (16%)	0 (0%)		8 (20%)	9 (23%)	0 (0%)		73 (37%)	17 (9%)	0 (0%)	
Low-grade positive (1, 2)	47 (21%)	51 (23%)	0 (0%)		9 (23%)	8 (20%)	0 (0%)		48 (24%)	34 (17%)	0 (0%)	
Negative	120 (55%)	135 (61%)	220 (100%)		23 (58%)	23 (58%)	40 (100%)		79 (40%)	149 (75%)	200 (100%)	
*Chest X-ray pattern*												
Fibrocalcific change	100 (46%)	80 (36%)	68 (31%)	0.006	13 (33%)	13 (33%)	8 (20%)	0.358	71 (36%)	67 (34%)	69 (35%)	0.915
Nodule or mass*	109 (50%)	98 (45%)	58 (26%)	< 0.001	29 (73%)	16 (40%)	13 (33%)	0.001	128 (64%)	134 (67%)	92 (46%)	< 0.001
Cavitation*	42 (19%)	22 (10%)	14 (6%)	< 0.001	4 (10%)	5 (13%)	2 (5%)	0.496	65 (33%)	30 (15%)	7 (4%)	< 0.001
Consolidation	118 (54%)	75 (34%)	108 (49%)	< 0.001	22 (55%)	22 (55%)	17 (43%)	0.434	118 (59%)	61 (31%)	76 (38%)	< 0.001
Bronchiectasis*	23 (11%)	116 (53%)	67 (31%)	< 0.001	11 (28%)	21 (53%)	20 (50%)	0.046	24 (12%)	122 (61%)	57 (29%)	< 0.001
Pleural effusion	18 (8%)	3 (1%)	15 (7%)	0.004	8 (20%)	2 (5%)	1 (3%)	0.014	21 (4%)	0 (0%)	7 (1%)	< 0.001
*Chest X-ray extent*												
Multifocal^a^	133 (61%)	141 (64%)	106 (48%)	0.002	28 (70%)	26 (65%)	17 (43%)	0.029	134 (67%)	151 (76%)	92 (46%)	< 0.001

NTM-LD, nontuberculous mycobacterial lung disease; TB, tuberculosis

^a^For the evaluation of the extent of lung involvement, each lung was divided into 3 areas. The pulmonary lesions with involvement in more than one lung area were regarded as multifocal

^*^p < 0.05 compared among training, internal test sets of the internal cohort, and the external test set of the external cohort

### Performance of DNN

**Fig. 3:**
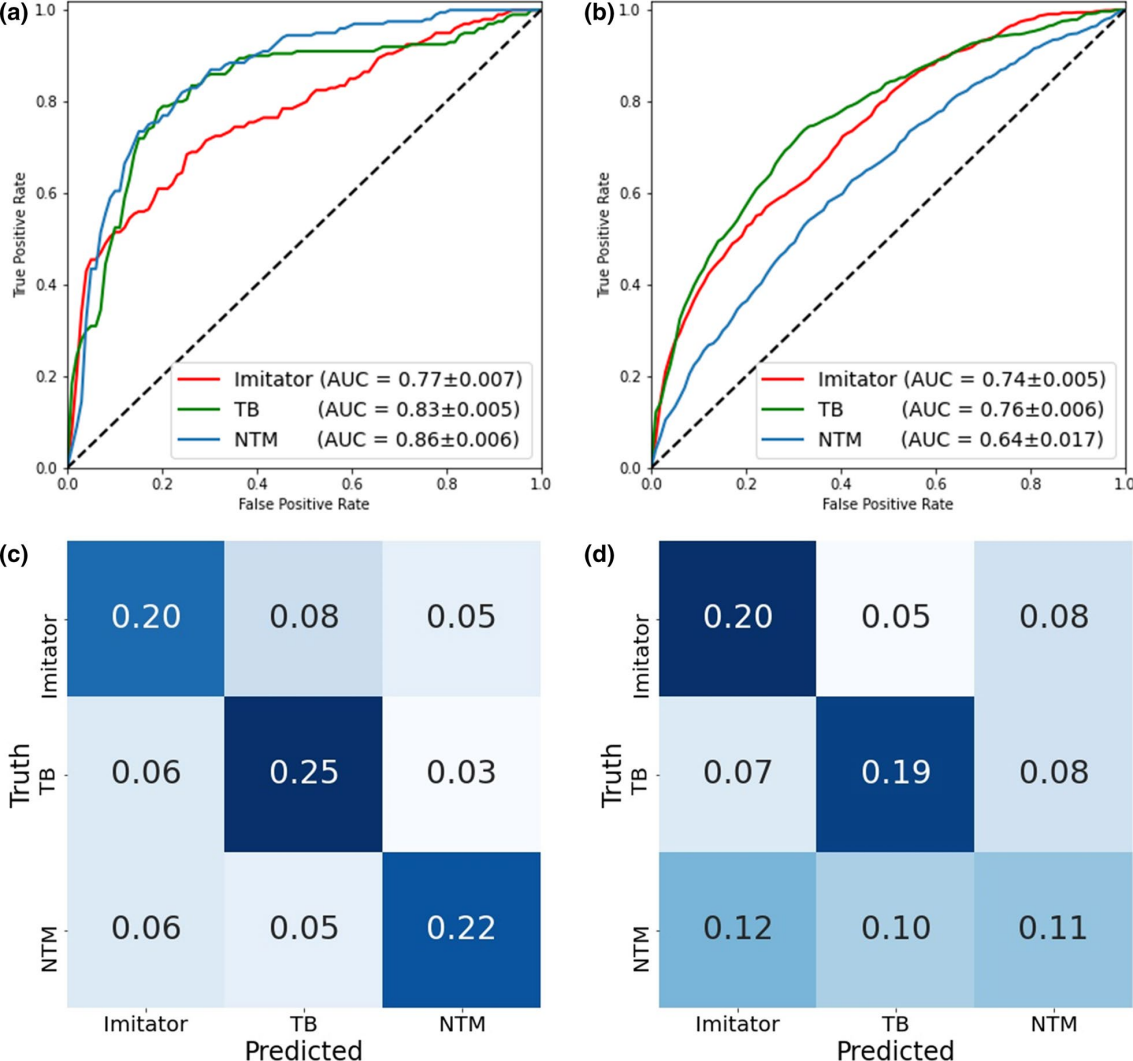
One-vs-others receiver operating characteristic (ROC) plots of our deep neural network (DNN) tested in the internal (**a**) and external (**b**) test sets are presented. Overall, the model showed acceptable generalizability for Imitator and tuberculosis (TB) predictions between the two tests. While the model was best at predicting non-tuberculous mycobacteria (NTM) in the internal cohort, it achieved the worst result in external cohort. This finding might come from great heterogeneity between NTM groups in the internal and external test sets. **c**, **d** demonstrates confusion matrices of DNN’s performance (**c**) and the pooled performance of the 12 pulmonologists (**d**) on the internal test set. The major distinction between human experts and machines can be found in NTM prediction. Even though the recruited pulmonologists are experts of mycobacterial diseases, they tended to make random guesses when chest X-rays (CXRs) of NTM were presented to them

Receiver operating characteristic (ROC) plots summarizing DNN performance on classifying mycobacterial lung diseases are illustrated in Fig. 3A and B. Our model achieved similar AUCs on each disease group. On the internal test set, it acquired AUCs of 0.83 ± 0.005 for TB, 0.86 ± 0.006 for NTM-LD, and 0.77 ± 0.007 for Imitator. When tested on the external test set, our model achieved AUCs of 0.76 ± 0.006 for TB, 0.64 ± 0.017 for NTM-LD, and 0.74 ± 0.005 for Imitator.

### Results of reader study

The individual diagnosis accuracy rate for each separate group was recorded in Table 2. The DNN model achieved a higher average accuracy rate of 66.5 ± 2.5% of the 3-class classification compared with human experts (49.2 ± 3.4%, *p* < 0.001). The 6 senior physicians achieved an average accuracy rate of 50.8 ± 3.0% (*p* < 0.001, compared to DNN) and the 6 junior physicians achieved an average accuracy rate of 47.5 ± 2.8% (*p* < 0.001, compared to DNN). When looking at the three individual groups, DNN has generally 18% more accurate cases on TB prediction (74.0% vs. 55.6%, *p* < 0.001) and is twice as accurate on NTM-LD detection (65.0% vs. 32.7%, *p* < 0.001) than physicians. By contrast, no significant difference was detected between physicians and our DNN on the Imitator prediction (59.2% vs. 60.6%, *p* = 0.816).

**Table 2:** Diagnostic accuracy of reader study

	Internal test				
	All doctors	Senior	Junior	DNN	p*
Overall	49.2 ± 3.4%	50.8 ± 3.0%	47.5 ± 2.8%	66.5 ± 2.5%	< 0.001
AFS					
AFS ( +)	46.8 ± 9.0%	47.1 ± 11.8%	46.6 ± 4.9%	73.0 ± 3.8%	< 0.001
AFS (-)	50.1 ± 5.4%	52.3 ± 5.4%	47.9 ± 4.4%	64.0 ± 2.9%	< 0.001
Diagnosis					
Imitator	59.2 ± 19.7%	61.2 ± 19.5%	57.1 ± 19.7%	60.6 ± 5.1%	0.816
TB	55.6 ± 13.5%	56.7 ± 8.0%	54.6 ± 17.3%	74.0 ± 6.7%	< 0.001
NTM-LD	32.7 ± 12.5%	34.6 ± 15.5%	30.8 ± 8.0%	65.0 ± 5.5%	< 0.001

DNN, deep neural network; AFS, acid-fast smear; TB, tuberculosis; NTM-LD, nontuberculous mycobacterial lung disease

p* compared between all doctors and DNN on diagnosis accuracy

Also, the relationship between predictions and the true diagnoses of the DNN (Fig. 3C) and pulmonologists (Fig. 3D) on the internal test set were presented as two confusion matrices. It is worth mentioning that human experts tended to equally allocate true NTM-LD cases into one of the three possible groups (36.4% [0.12/0.33] as Imitator, 30.3% [0.10/0.33] as TB, and 33.3% [0.11/0.33] as NTM-LD). Finally, to realize individual variance of prediction on the same cases, we further examined the inter-rater correlation coefficient (ICC) of physicians and DNNs (Additional file 1: Appendix E). On the internal test set, the ICC score is 0.244 (95% Confidence interval [CI]: 0.188–0.312) of the 12 physicians and 0.799 (95% CI: 0.754–0.841) of our DNNs.

### Class activation heatmap

**Fig. 4:**
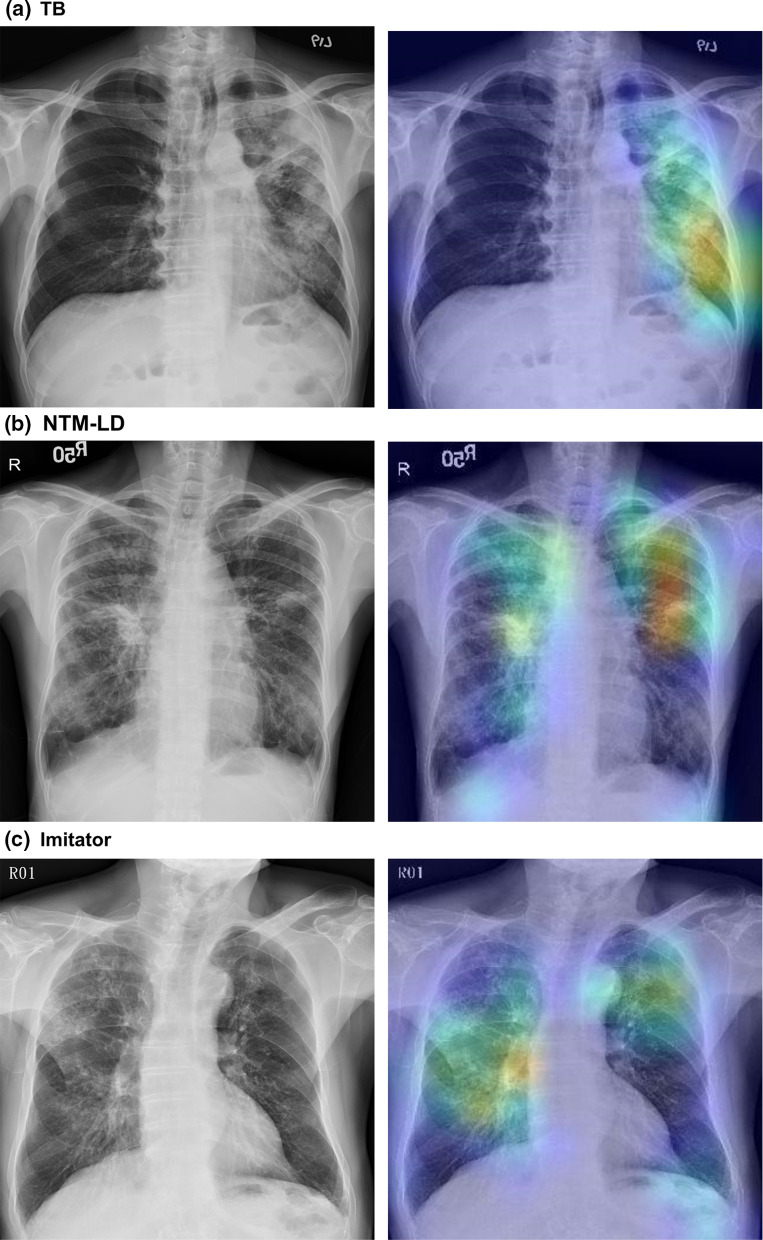
Chest radiography and the class activation heatmaps for tuberculosis (TB), nontuberculous mycobacterial lung disease (NTM-LD), and Imitators. The colours on the heatmap represent the diagnostic weights of determining the class in interest. The hotter the colours are (red and yellow), the more important the areas are to the final decision in the deep neural network (DNN). **a** demonstrates patchy and poorly defined consolidation with cavities at the left upper and lower lobes that are highly suggestive for pulmonary TB. In **b**, the chest radiography reveals bilateral bronchiectasis with nodular infiltrations, which termed nodular bronchiectasis is a typical presentation of NTM-LD. **c** shows bronchiectasis at bilateral lung fields

Figure 4 demonstrates three activation heatmaps for TB, NTM-LD, and Imitator, respectively. In these three representative cases, the DNN model correctly localized lesions and classified the CXRs into TB, NTM-LD, and Imitator, respectively.

### DNN as a screening tool in different mycobacteria prevalence

Among different TB prevalence in “TB test”, our model has stable performance in terms of AUC (0.77–0.77), sensitivity (0.62–0.66), and specificity (0.77–0.78) (Table 3). Using DNN as a screening tool for TB detection could save 77%, 76%, and 72% of further tests with a total loss of 2%, 2%, and 6% of confirmed TB cases among low, medium and high TB prevalence, respectively.

**Table 3:** Comparison between different deep neural network models on tuberculosis (A) and mycobacteria (B) detection

	Our DNN model			DNN model analogue		
	Low TB burden	Medium TB burden	High TB burden	Low TB burden	Medium TB burden	High TB burden
*(A)*						
AUC	0.77 ± 0.14	0.77 ± 0.11	0.77 ± 0.06	0.76 ± 0.11	0.76 ± 0.09	0.77 ± 0.06
Sensitivity	0.62 ± 0.23	0.66 ± 0.20	0.66 ± 0.12	0.64 ± 0.22	0.65 ± 0.16	0.66 ± 0.11
Specificity	0.78 ± 0.04	0.78 ± 0.04	0.77 ± 0.04	0.82 ± 0.03	0.84 ± 0.03	0.84 ± 0.04
PPV	0.09 ± 0.04	0.14 ± 0.04	0.30 ± 0.05	0.11 ± 0.05	0.18 ± 0.05	0.38 ± 0.07
NPV	0.98 ± 0.01	0.98 ± 0.01	0.94 ± 0.02	0.98 ± 0.01	0.98 ± 0.01	0.95 ± 0.02
Reduced further test (%)	77 ± 4	76 ± 4	72 ± 4	81 ± 3	82 ± 3	78 ± 3
Number needs to screen	12.67 ± 5.93	8.33 ± 3.87	3.47 ± 0.57	10.03 ± 4.91	5.98 ± 2.02	2.77 ± 0.62
Misclassified NTM-LD as TB (%)	14 ± 7	16 ± 16	14 ± 15	31 ± 10	36 ± 21	32 ± 21
*(B)*						
AUC	0.77 ± 0.05	0.76 ± 0.07	0.74 ± 0.05	0.67 ± 0.06	0.71 ± 0.07	0.73 ± 0.07
Sensitivity	0.79 ± 0.08	0.78 ± 0.12	0.76 ± 0.09	0.38 ± 0.10	0.49 ± 0.13	0.55 ± 0.11
Specificity	0.61 ± 0.04	0.59 ± 0.05	0.60 ± 0.05	0.85 ± 0.03	0.85 ± 0.04	0.85 ± 0.03
PPV	0.34 ± 0.03	0.18 ± 0.03	0.28 ± 0.03	0.39 ± 0.08	0.27 ± 0.08	0.43 ± 0.08
NPV	0.92 ± 0.03	0.96 ± 0.02	0.93 ± 0.03	0.85 ± 0.02	0.94 ± 0.02	0.91 ± 0.02
Reduced further test (%)	53 ± 4	56 ± 5	54 ± 4	80 ± 3	82 ± 4	79 ± 3
Number needs to screen	2.99 ± 0.30	5.81 ± 1.11	3.67 ± 0.47	2.70 ± 0.60	4.07 ± 1.45	2.40 ± 0.49
Misclassified TB as imitator (%)	26 ± 21	26 ± 17	26 ± 11	38 ± 24	33 ± 19	37 ± 14
Misclassified NTM-LD as imitator (%)	20 ± 8	18 ± 15	21 ± 17	67 ± 10	70 ± 19	68 ± 19

DNN, deep neural network; TB, tuberculosis; AUC, area under the receiver operating characteristic curve; PPV, positive predictive value; NPV, negative predictive value; NTM-LD, nontuberculous mycobacterial lung disease

On the other hand, in “Mycobacteria test”, our model also has stable AUC (0.74–0.77), sensitivity (0.76–0.79), and specificity (0.59–0.61) in the setting of different TB prevalence. Using DNN as a screening tool for mycobacteria detection could save 53%, 56%, and 54% of further tests with a total loss of 8%, 4%, and 7% of confirmed cases with mycobacterial lung disease among low, medium and high TB prevalence, respectively.

### Performance of our DNN model and the DNN model analogue

Table 3 summarizes the screening performance of our DNN and the DNN model analogue in “TB and Mycobacteria test”. Compared to the DNN model analogue, our model has similar performance in terms of AUC (0.77–0.77 vs. 0.76–0.77) and sensitivity (0.62–0.66 vs. 0.64–0.66), but lower specificity (0.77–0.76 vs. 0.82–0.84) in “TB test”. Although our model reduces less patients needing further tests (72–77% vs. 78–81% using the DNN model analogue), it significantly avoids incorrectly predicting NTM-LD patients as TB (misclassification rate: 14–16% vs. 31–36%).

On the other hand, in “Mycobacteria test”, our model has similar AUC (0.74–0.77 vs. 0.67–0.73), better sensitivity (0.76–0.79 vs. 0.38–0.55) but worse specificity (0.59–0.61 vs. 0.85–0.85) compared to the DNN model analogue. After breaking down, our model consistently has lower misclassification rate on incorrectly predicting TB as Imitator (26–26% vs. 33–38%) and wrongly predicting NTM as Imitator (18–21% vs. 67–70%).

## Discussion

Our study revealed that the deep learning algorithm was able to distinguish TB and NTM-LD patients by CXRs and significantly outperformed experienced pulmonologists. Also, we demonstrated that our model was capable of providing consistent performance even in environments with different mycobacteria prevalence and had significantly lower misclassification rate for patients with clinical suspicion of mycobacterial lung disease. These observations provide more solid grounds for future roles that DNN-based models may play for mycobacterial disease management in clinical practice and public health.

In the past, much attention has been placed on pulmonary TB, which has led to under-recognition of NTM-LD [21]. Many patients with NTM-LD have received empiric treatment for TB until culture result available [22]. However, NTM are often resistant to many of the first- and second-line anti-TB drugs [23]. Inappropriate treatment for NTM-LD might place the patient at increased risk for developing drug-resistant infections, which could carry a dismal outcome [24]. Additionally, falsely presumptive diagnosis usually causes unnecessary airborne isolation and prolonged hospitalization and leads to waste of medical resources [25].

Our study provides a new solution to meet these clinical needs. The model outperformed participating physicians and was robust under different circumstances even though it was widely perceived that no radiographic characteristics could reliably distinguish NTM-LD from pulmonary TB [26]. Furthermore, we decoded the rationale inside the DNN with visualization heatmaps, which can help future physician users either discover undetected lesions or deny impossible decisions made by the DNN. Performance-wise, our model acquired AUCs of 0.83 and 0.86 for recognizing TB and NTM-LD, which are comparable to the state-of-the-art study using chest CT images and achieving an AUC for differentiating NTM-LD from TB [21]. However, given the better accessibility, lower-cost, and faster image processing time, we argue that our approach using CXRs can provide better assistance for clinicians as a first-line screening tool.

Special attention, however, should still be paid to the generalizability of DNN models, especially when patient population and characteristics differ geographically. In our study, for instance, the drop of the DNN model performance from internal to external cohort may result from the difference of patient age, mycobacteria load and radiographic patterns between cohorts, especially in the NTM-LD group (comparing with NTM-LD in the internal test set, NTM-LD in the external test set were younger (*p* = 0.001), more likely to be acid-smear negative (*p* = 0.023), having nodule or mass (*p* = 0.001), having consolidation (*p* = 0.003) and having pleural effusion (*p* = 0.027)). In practical application, a possible solution to the decreased performance of the model in external cohort is to use a small number of images in external cohort to fine-tune the model (see Additional file 1: Appendix C).

This study also puts emphasis on the presence of NTM patients in developing models. Compared to the “DNN model analogue”, our model showed a lower rate of misclassifying NTM patients to TB in the “TB analysis”. This finding highlights the potential of DNN-based model as a screening tool for reducing unnecessary airborne isolation and inappropriate treatment among patients with NTM-LD. In addition, our model was more resistant to misclassifying the highly mimicking Imitator while the DNN model analogue fails to provide satisfactory sensitivity rate in ‘mycobacteria test’. Therefore, even though a model is only developed for identifying TB patients from other common lung diseases, we suggest that the developer should still consider including NTM-LD patients in the training set given its non-negligible presence in the real world.

Another major strength of this study is the inclusion of the Imitator group as our control. Several past studies have shown the potential of applying deep learning models to assist TB screening. Most of the study used relatively healthy patients as their control group against the TB patients for screening [11, 12]. In real-world clinical practice, however, physicians need to distinguish TB and NTM-LD from multiple mimicking diseases such as structural lung diseases with secondary bacterial infection, cavitating lung cancer, or chronic pneumonia [27–29]. Therefore, we decided to include the Imitator group and made a more challenging but commonly faced test setting, for both physicians and our DNN.

Our study also has limitations. First, we did not include a healthy control group. We, however, also considered this as a major distinction of our study since previous studies have already demonstrated that discriminating between CXRs of healthy and TB participants is not difficult for DNN. Secondly, patients with TB and NTM-LD co-infection were not enrolled in this study. Nevertheless, the incidence rate of NTM–TB coinfection was relatively low in real world [30]. Thirdly, the reader study was based on pulmonologists rather than experienced radiologists. Furthermore, we did not provide extra training cases to pulmonologists before the reader study since these physicians have been diagnosing and treating patients with suspects of mycobacterial lung diseases in their routine clinical practice. Therefore, the accuracy metric might be misleadingly low for pulmonologists. Lastly, the reader study was only conducted on the 120 CXRs in the internal test set. We could not exclude the possibility that pulmonologists may achieve better or even surpass the DNN in terms of classification accuracy on the external test set, especially considering the decline in performance of the DNN model on the external test set.

## Conclusion

In conclusion, we demonstrate that our DNN model is more accurate than experienced physicians on classifying suspects of mycobacterial diseases and can robustly reduce the requirements for further confirmation test. These results indicate that DNN-based models could potentially become great first-line screening tools to compensate for physicians and unload them from diagnosing and differentiating mycobacterial lung diseases.

## Supplementary Information

**Additional file 1**. Supplementary material.

## Data Availability

The datasets used and/or analyzed during the current study are available from the corresponding author on reasonable request.

## Abbreviations

AFS: Acid-fast smear
ANOVA: One-way analysis of variance
AUC: Area under the receiver operating characteristic curve
CI: Confidence interval
CT: Computed tomography
CXP: CheXpert
CXR: Chest X-ray
DNN: Deep neural network
ICC: Inter-rater correlation coefficient
MMC: MIMIC
NTM: Non-tuberculous mycobacteria
NTM-LD: Nontuberculous mycobacterial lung disease
TB: Tuberculosis
TB-PCR: Nucleic acid amplification test for *Mycobacterium tuberculosis* complex
