# Supplementary material for: A deep learning model using chest X-ray for identifying TB and NTM-LD patients: a cross-sectional study
Source: Insights Imaging. 2023 Apr 15;14:67. doi: 10.1186/s13244-023-01395-9 (PMC10105818; doi:10.1186/s13244-023-01395-9)
Supplement: Supplementary file 1 — Additional file 1. Supplementary material. [file 13244_2023_1395_MOESM1_ESM.pdf]

<u>ELECTRONIC SUPPLEMENTARY MATERIAL</u>

A deep learning model using chest X-ray for identifying TB and NTM-LD patients: a cross-sectional study

Appendix A. Transfer learning

In this study, we leveraged transfer learning to get better models. To begin with, we used two large public CXR databases, MIMIC and CheXpert, to pretrain DenseNet121 [1] (**Figure 2A**). These two databases contain 14 commonly seen CXR diagnoses but do not include mycobacterial diseases. Since DenseNet121 was calibrated to learn radiological diagnoses, we assumed that the image representations outputted by DenseNet's encoder had "radiological knowledge" to some extent; therefore, they were useful for learning mycobacterial predictions.

We then froze DenseNet's encoder, put two new dense layers on top of it, and used our in-house mycobacterial datasets to train the model. Conceptually, the frozen encoder would extract "radiological features" from mycobacterial images and fore-propagate it to the following dense layers. The dense layers were then trained to learn specific diagnoses for the three mycobacterial diseases (the "final model"). In **Appendix C**, we also tested if fine-tuning our final model on external cohort could improve performance. Please refer **Appendix C** to see more details.

Appendix B. Hyperparameter choices of our DNN

DenseNet121 was used as the backbone of all our DNN models. We began our study by training DenseNet121 on the MIMIC and CheXpert datasets. Adam optimizer was selected to find convergence and the empirically best learning rate (0.001) was kept for the rest of this study.

After the pretraining, two fully connected dense layers consisting of 512 neurons were added on top of the DenseNet121 backbone. We used 128 images for a single batch since this was the largest number allowed under our Google Colab setting. To compute the standard deviation of the performance, the above process with identical hyperparameter selections was kept for the 12-time repetition. We chose to produce 12 copies of the model to pair with the 12 pulmonologists recruited in this study.

Appendix C. Test performance after fine-tuning on the external cohort

We randomly sampled 300 patients out from the external cohort and used them to fine-tune the model trained in the internal cohort. This led to 300 patients remaining for testing **(Figure A1)**. The characteristics of patients in this fine-tuning and external validation sets can be found in **Appendix D**. After fine-tuning, our model achieved similar performance in the Imitator (AUC = 0.77 vs. 0.78 in internal and

external test sets respectively) and in the TB (AUC = 0.83 vs. 0.82 respectively) groups. The major difference between internal and external test performance was observed in the NTM (non-tuberculous mycobacteria) group AUC = 0.86 vs. 0.73 respectively), but the difference was less than model without fine-tuning.

Figure A1.

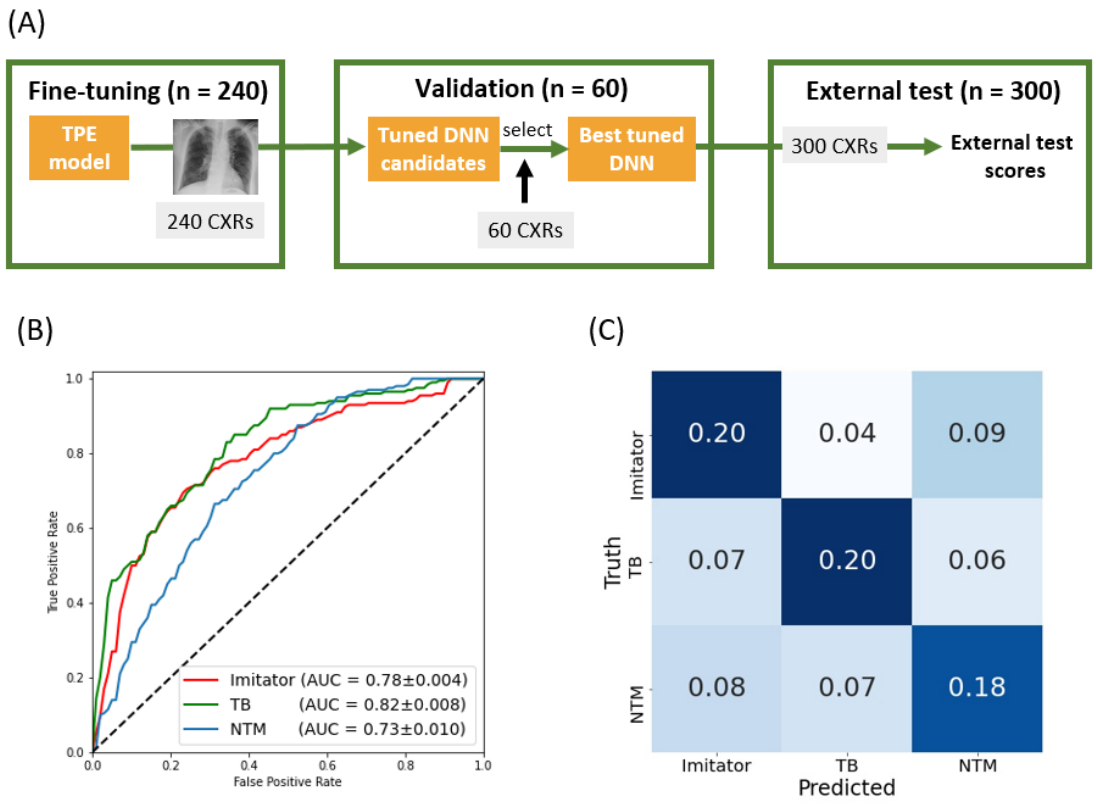

(A) Similar to developing the model in the internal cohort, we split the external cohort to fine-tuning, validation, and test sets. Since the model has learned some mycobacterial chest x-rays (CXRs) from the internal cohort, we reduced the number of CXRs in the fine-tuning set compared to its first training run.

(B) The model achieved similarly great performance after using fewer CXRs to fine-tune. Along with Figure 3. (A) (B), although the model will lose some capability when applying to an unseen dataset, it can be calibrated using few samples and retrieve reasonably good performance.

(C) Even after fine-tuning, the model still made more mistakes in differentiating non-tuberculous mycobacteria (NTM) from Imitator. This proves the confusing nature of Imitator and justifies our motivation of including Imitator in our study.

Appendix D. Clinical characteristics of patients with presumptive mycobacterial lung diseases

	External cohort, fine-tuning (n=240)			External cohort, validation (n=60)			Internal cohort, validation (n=120)		
	TB (N=80)	NTM-LD (N=80)	Imitator (N = 80)	TB (N=20)	NTM-LD (N=20)	Imitator (N=20)	TB (n=40)	NTM-LD (n=40)	Imitator (n=40)
Age (years)	54.6±21.1	66.1±16.2	67.7±11.8	55.5±19.8	64.1±15.5	64.7±14.0	63.6±19.2	65.3±12.8	69.4±11.7
Male, n (%)	56 (70%)	45 (56%)	31 (39%)	14 (70%)	10 (50%)	12 (60%)	29 (73%)	16 (40%)	17 (43%)
**Acid-fast smear**									
High-grade positive (3, 4)	30 (38%)	4 (5%)	0 (0%)	8 (40%)	1 (5%)	0 (0%)	8 (20%)	6 (15%)	0 (0%)
Low-grade positive (1, 2)	23 (29%)	10 (13%)	0 (0%)	5 (25%)	3 (15%)	0 (0%)	15 (38%)	8 (20%)	0 (0%)
Negative	27 (34%)	66 (83%)	80 (100%)	7 (35%)	16 (80%)	20 (100%)	17 (43%)	26 (65%)	40 (100%)
**Chest X-ray pattern**									
Fibrocalcific change	32 (40%)	23 (29%)	19 (24%)	8 (40%)	9 (45%)	8 (40%)	15 (38%)	11 (28%)	13 (33%)

Nodule or mass	49 (61%)	47 (59%)	33 (41%)	16 (80%)	14 (70%)	8 (40%)	23 (58%)	17 (43%)	18 (45%)
Cavitation	27 (34%)	8 (10%)	3 (4%)	6 (30%)	3 (15%)	1 (5%)	5 (13%)	0 (0%)	1 (3%)
Consolidation	45 (56%)	31 (39%)	30 (38%)	8 (40%)	7 (35%)	8 (40%)	19 (48%)	15 (38%)	18 (45%)
Bronchiectasis	6 (8%)	49 (61%)	24 (30%)	5 (25%)	9 (45%)	5 (25%)	5 (13%)	22 (55%)	15 (38%)
Pleural effusion	11 (14%)	0 (0%)	5 (6%)	1 (5%)	0 (0%)	0 (0%)	11 (28%)	1 (3%)	2 (5%)
**Chest X-ray extent**									
Multifocal	54 (68%)	58 (73%)	33 (41%)	13 (65%)	15 (75%)	8 (40%)	24 (60%)	21 (53%)	18 (45%)

Abbreviation: NTM-LD, nontuberculous mycobacterial lung disease; TB, tuberculosis

Appendix E. The inter-rater correlation coefficient (ICC) score for each rater group

	ICC score (95% Confidence interval)
**All doctors**	0.244 (0.188 - 0.312)
**Senior physicians**	0.244 (0.154 - 0.307)
**Junior physicians**	0.244 (0.172 - 0.328)
**DNN**	0.799 (0.754 - 0.841)

Abbreviation: DNN, deep neural network

References

1. Huang G LZ, Van Der Maaten L, Weinberger KQ (2017) Densely Connected Convolutional Networks. Proceedings of the IEEE Conference on Computer Vision and Pattern Recognition.4700-8.

2. Gao Y, Cui Y (2020) Deep transfer learning for reducing health care disparities arising from biomedical data inequality. Nat Commun.11(1):5131. https://doi.org/10.1038/s41467-020-18918-3.
